# Incognito standardized patients for studying healthcare stigma: partial deception and other ethical challenges

**DOI:** 10.1186/s12910-026-01397-4

**Published:** 2026-03-09

**Authors:** Siyan Meng, Yunqing Fei, Danyang Luo, Sean Y. Sylvia, M. Kumi Smith

**Affiliations:** 1 Department of Health Behavior, Society and Policy, School of Public Health, Rutgers University, Piscataway, NJ USA; 2 Center for Global Health and Social Responsibility, University of Minnesota Twin Cities, Minneapolis, MN USA; 3 Department of Behavioral, Social, and Health Education Sciences, Rollins School of Public Health, Emory University, Atlanta, GA USA; 4 Department of Health Policy & Management, University of North Carolina, Chapel Hill, NC USA; 5 Division of Epidemiology & Community Health, University of Minnesota Twin Cities, 1300 South Second Street, Suite 300, Minneapolis, MN 55454 USA

**Keywords:** Standardized patient method, Ethical considerations, HIV, Enacted stigma, Marginalized populations

## Abstract

This paper examines the ethical considerations of using the incognito clinic visits by standardized patients (SP) in order to measure enacted stigma in healthcare settings. It reviews traditional SP methods, introduces the new method, and discusses its potential harms and benefits.

Incognito (or “unannounced”) standardized patient (SP) visits are a common technique for measuring healthcare quality in health services research. Trained actors present standardized disease cases to medical providers to observe their clinical behaviors. Because providers are not told when visits will take place, incognito SPs provide windows of insight into provider behaviors in real clinical settings. It also addresses longstanding measurement challenges including low willingness to self-report undesirable behaviors or tendencies to alter behavior under observation (Hawthorne effect [1]). Incognito SP techniques have been used to assess care quality in medical domains, including diagnostic accuracy [2], prescribing behaviors [3], and health communications [4].

In this commentary, we describe a unique application of the incognito SP method in which we use it to measure enacted healthcare stigma, or discrimination by healthcare workers toward patients. We achieve this by combining incognito SPs with experimental audits in which SP attributes are randomly varied across case presentations. Our audit design allows us to compare how providers interact with different patient identities—e.g., HIV status or sexual orientation [5]—to yield the erosion of healthcare quality due to HIV stigma or homophobia. Past uses of experimental audits—mostly by sociologists—have famously uncovered discrimination in employment [6], housing [7], and social encounters [8] in the US. In prior studies, interactions typically involve casual, real-world encounters between actors and unsuspecting individuals, as in one study that compared employer callback rates for fictitious resumes with stereotypically Black- versus White-sounding names [6]. These studies often involve partial deception, ethically justified by the anonymity of encounters and the minor risks to participants relative to the substantial harms of discrimination [9, 10].

Our SP-based experimental audit features several factors that raise new ethical questions. For one, our SPs interact with medical providers who are formally enrolled as study participants rather than through chance encounters with strangers. We analyze the resulting stigma at the group level, but a data breach could therefore potentially link problematic behaviors to individual providers. In addition, the audits were used to measure stigma as the primary outcome in a randomized trial evaluating a provider training program. To maintain rigor and objectivity of the measure—and to reduce participant vigilance toward SP encounters—we avoided explicit references to “HIV” or “sexual orientation” during recruitment, instead describing the study as focused on “patient–provider communication with marginalized patients.” Hereafter, we analyze the unique challenges of the fact that SP–provider interactions take place in non-public settings (i.e. clinics) and our use of partial disclosure of study aims. We also consider potential solutions to guide ethical use of the incognito SP method for health equity research.

Potential harms of our design stem from collecting individual-level data on consented providers who are not fully informed that they are being assessed for stigmatizing care. Beyond the typical harms of deception—e.g., psychological harm, erosion of trust in research—lies the risk that a data breach could uncover problematic behaviors that could impair providers’ job security or reputation. Although this may constitute greater-than-minimal risk, overall risk can still be considered minimal if frequency is sufficiently low. Designs should therefore mitigate this risk by explicitly disclosing potential consequences of a data breach during consent procedures. Additionally, teams must consider the potential research gains against potential damage to healthcare access among real patients who may have to compete with SPs for appointment slots.

Potential benefits that are novel to our study design include the added value of SP-collected stigma measures that improve understanding of stigma, which can, in turn, unlock new strategies for addressing it. Although a substantial body of research has developed and tested many stigma reduction interventions [11], their design largely relies on theoretical reasoning to specify the underlying causes—and solutions—of stigma due to their inability to observe the very behaviors they seek to change. The promise of training informed by SP data stems from the fact that they target real service gaps observed in clinical practice. Second, incognito SP visits can yield novel scientific knowledge about stigma. For example, our work has shown ways in which healthcare stigma today manifests less as the verbal or physical abuse reported in earlier eras of HIV research [12, 13] and more often as avoidance (e.g., more perfunctory visits) or neglect (e.g., less thorough exams, less history taking). These findings advance scientific understanding by revealing diverse, indirect forms of stigma, new mechanisms of harm, and potential strategies for intervention.

A final consideration is SP safety. Although not formally assessed in ethical reviews, SPs may face risks such as negative treatment from providers or the risk of SP visit data being conflated with their own medical records. These risks can be particularly worrisome if SPs belong to marginalized groups like gay or bisexual men as in our study. In our experience, such risks are best mitigated through formal SP safety protocols that aim to protect SPs with standards commensurate of research participant protections. Our SP protocols were developed with input from the simulation literature [14] and input from our two community advisory boards (CAB) of providers and LGBTQ+ community members, which dictated methods for recruitment, training, and monitoring of SP well-being. Though we did not formally evaluate the impact of our SP safety protocols, qualitative interviews with a subset of our SPs revealed that many found special value in the role, which they described as a novel opportunity to participate in scientific research as change agents rather than study subjects [15].

## Conclusion

Our adaptation of the incognito SP method to measure healthcare stigma highlights new but largely familiar ethical challenges, including data security and partial deception. While additional safeguards are essential, researchers must weigh potential harms against anticipated benefits. In our study, this assessment was integral to the ethical approval process and informed by extensive consultation with community advisory boards and university IRB analysts, shaping our recruitment, consent, and data collection procedures. Ultimately, these risks must be balanced against the significant scientific insights such methods can offer, with the potential to advance more equitable, stigma-free healthcare.

## Data Availability

No datasets were generated or analysed during the current study.

## Abbreviations

SP: Standardized patient
HIV: Human immunodeficiency virus
IRB: Institutional review board
CAB: Community advisory board
